# The association between diabetes and cognitive changes during aging

**DOI:** 10.1080/02813432.2020.1802140

**Published:** 2020-08-10

**Authors:** Sanna Papunen, Anna Mustakallio-Könönen, Juha Auvinen, Markku Timonen, Sirkka Keinänen-Kiukaanniemi, Sylvain Sebert

**Affiliations:** a Centre for Life Course Health Research, Faculty of Medicine, University of Oulu, Oulu, Finland; b Biocenter Oulu, University of Oulu, Oulu, Finland; c Department of Genomics of Complex Diseases, School of Public Health, Imperial College London, London, UK

**Keywords:** Diabetes, elderly, cognitive functions, decline, systematic review

## Abstract

**Background:**

Worldwide, we are observing a rising prevalence of dementia and mild cognitive impairments that often co-occur with the heightened incidence of non-communicable diseases in the elderly. It is suggested that type 2 diabetes and defects in glucose metabolism might predispose to poorer cognitive performances and more rapid decline in old age.

**Methods:**

to address existing knowledge gaps in this area, we systematically reviewed the literature to identify whether patients with type 2 diabetes (T2DM) and pre-diabetes are at a higher risk of poorer cognitive performance, and whether the risk (if any) might affect specific cognitive abilities. We concentrated the review on elderly individuals (65 years or older) at intake. In total, 3251 original articles were retrieved, of which 17 met our inclusion and quality control criteria, which comprised 12 structured questions used to define the articles.

**Results:**

11 of 17 studies found a statistically significant decline in cognition among individuals who had T2DM or pre-diabetes compared to their non-diabetic counterparts. The association between diabetes and cognitive decline was not always clear, and the extent of the cognitive tests used seemed to have the greatest effect on the results.

**Conclusion:**

Focusing on a population age 65 years and over, we found insufficient evidence to support an association between pre-diabetes stages and mild cognitive impairment. However, there is consistent evidence to support diabetes as an independent risk factor for low cognitive ability in the elderly. Finally, we found insufficient evidence to support effect of T2DM on distinct cognitive ability due to the scarcity of comparable findings.

## Introduction

According to World Alzheimer report by ADI (Alzheimer`s Disease International) in 2019, it was estimated that 50 million people worldwide were living with dementia, and that the estimate might increase to 152 million by 2050. The total costs for health care, long-term care, and hospice care for individuals with dementia are substantial [1]. Since there is no effective cure for dementia, it is important to focus instead on preventing the cognitive decline that may subsequently lead to dementia.

In midlife smoking, obesity, diabetes, high cholesterol and hypertension are known to be risk factors for cardiovascular diseases, but they are also often suspected to be associated with a higher risk of cognitive decline and dementia. Unlike genetic risk factors, these cardiovascular disease risk factors can be often mitigated that the likelihood of developing either cardiovascular diseases or cognitive decline and dementia decreases.

Several studies have suggested T2DM could be causally associated with cognitive impairments through vascular pathophysiological mechanisms, and longitudinal findings have indicated that T2DM can influence accelerated cognitive decline [2]. Neurodegeneration and cognitive decline have been observed among insulin- resistant patients [3]. Recent studies have helped reveal the connection between insulin function in the brain and cellular and molecular pathogenesis between T2DM and cognitive dysfunction. [4,5] Along with insulin resistance vascular endothelial dysfunction, inflammation and blood-brain barrier injury, demyelination and axonal loss are suggested to accelerate neurodegeneration in patients with T2DM [6].

This systematic review focused on the relation between impaired glucose tolerance or diabetes and changes in cognitive performance during aging. We selected studies that included the elderly (>65 years), who are typically already past their working career and are rarely specifically targeted in prevention programs, to generate evidence for a direct link between defects in glucose metabolism and the risk of unhealthy aging. This review was aimed at yielding a better understanding of factors affecting the heterogeneity of the finding and employed sensitivity analysis to explore long-term effect and potential specificity of the findings in the affected cognitive dimensions.

## Methods

### Literature search

We systematically examined the literature, aiming to include all original articles in English language that examined the relation between type 2 diabetes and/or pre-diabetes with cognitive decline in the elderly. The literature search was first performed using Scopus as the database on 6 May 2017. We chose to use only Scopus, because PubMed and Medline (Ovid) content is a subset of Scopus. We applied the following search criteria, in which * represents zero or more arbitrary characters: ‘cogniti*’, ‘diabet* or glucose’, ‘aged or aging’ and ‘follow-up or longitudinal or cohort’. Search results were retrieved from Scopus database by using the application programming interface available for the Python programming language and were then imported into Microsoft Excel for further manual inspection.

A second search was conducted on 4 May 2019 with the same methodology to update the literature review with the newest and most relevant articles. The search resulted in 3251 articles in total.

### Inclusion and exclusion criteria

We included only population-based original articles that were written in English. The articles had to meet the following criteria

- contain information on type 2 diabetes or pre-diabetes at baseline;
- contain information on cognitive function or dementia;
- include an elderly population, defined as being over 65 years of age at the start of the study;
- published, in English language, as a peer-reviewed article within the last 20 years;
- include only human;
- follow ethical regulation;
- include at least one follow-up evaluation of type 2 diabetes or pre-diabetes at baseline;
- be an observational study or Randomized Controlled Trial;
- exclude of mild cognitive impairment or dementia at baseline.

We excluded clinical study, randomized control trials and studies including patients with type 1 diabetes or patients whose condition could not be differentiated from T2D.

### Study selection

**Figure 1.**
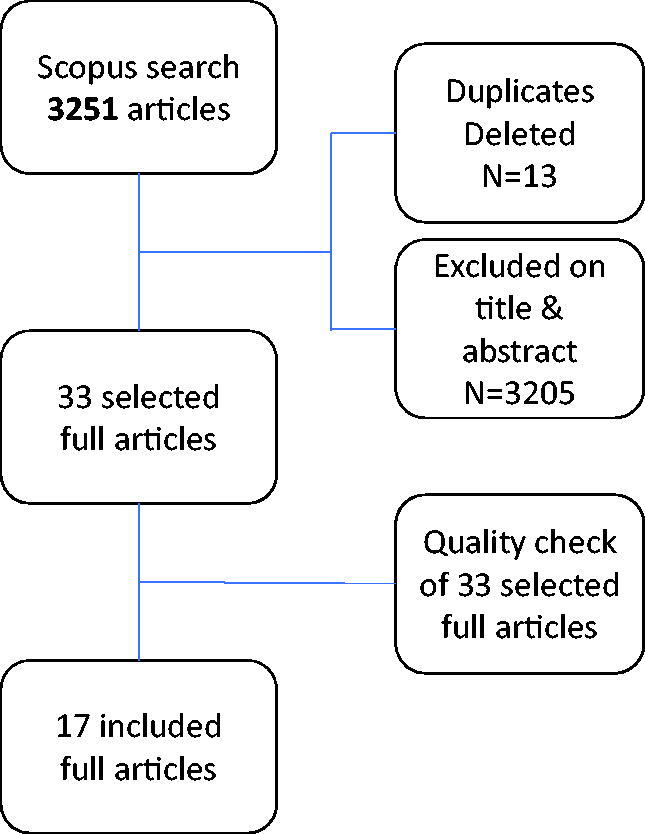
A flow chart including a detailed description of the research process..

Titles and abstracts were scanned manually on relevance, and potentially eligible papers were collected in full-text versions. Duplicates were screened and excluded. The search resulted in 33 articles. These articles were read by two independent readers (AM-K, SP) in order to determine whether they met the inclusion criteria (Figure 1).

### Definition of prediabetes

WHO criteria for prediabetes are fasting blood glucose level 6.1–6.9 mmol/l (IFG; impaired fasting glucose) or 2-h glucose level 7.8–11 mmol/l (IGT; impaired glucose tolerance). Now reviewed studies that reported results concerning prediabetes and cognitive changes defined prediabetes according to fasting blood glucose level, random blood glucose level or A1C (Hba1c) measurement.

### Definition of type 2 diabetes

Studies (see Table 1) meeting the criteria to define type 2 diabetes were considered in this review. These included self-reported use of hypoglycemic medication or diabetes diagnosed by a medical practitioner or clinical definitions based on fasting glucose level measurement, glycated hemoglobin A1C (Hba1c) measurement and/or oral glucose tolerance test. The clinical definitions were following WHO criteria for diabetes i.e. fasting glucose level ≥7 mmol/l or 2-h blood glucose level >11 mmol/l, HbA1c ≥6.5% (48 mmol/mol); or a random blood glucose ≥ 11.1 mmol/l.

**Table 1.** Characteristics of included articles.

Authors	Year of publication	Study name	Country	Ethnic groups	*N* at inclusion	Age at inclusion	Length of follow-up	Loss of follow-up
Altschul et al. [14]	2018	Lothian Birth Cohort of 1936 (LBC1936)	UK	Caucasian	1091	>68	13 years	543
Bangen et al. [13]	2015	Washington Heights – Inwood Columbia Aging Project (WHICAP)	USA	American	1493 + 448	>65 years	6 years	448
Espeland et al. [16]	2011	The Women’s Health Initiative Study on Cognitive Aging (WHISCA)	USA	American	2304	>65 years	5 years	141
Formiga et al. [11]	2014	Octabaix Study	Spain	Spanish, Caucasian	328	>85 years	2 years	167
Hassing et al. [21]	2004	Origins of variance in the old-old (OCTO-Twin Study)	Sweden	Caucasian	274	>80 years	6 years	–
Logroscino et al. [17]	2004	The nurses’ health Study	USA	American	18999	>70 years	2 years	2403
Marseglia et al. [10]	2019	Swedish National Study on Aging and Care-Kungsholmen (SNAC-K)	Sweden	Caucasian	2746	>60	median 6.4 years	1104
Mayeda et al. [27]	2015	The Sacramento Area Latino Study on Aging (SALSA)	USA	Latino	1789	>60 years	Median 7.6 years	707
Okereke et al. [18]	2008	The Physicians’ Health Study and the Women’s Health Study	USA	American	12330	>65 years	2 years	Men 714 Women 685
Pappas et al. [19]	2016	HRS (the Health and Retirement Study)	USA	Caucasian, African American	4419	>65 years	6 years	–
Rouch et al. [7]	2012	The PROOF study	France	Caucasian	1100	>65 years	8 years	937
Samaras et al. [8]	2014	The Sydney Memory and Ageing Study	Australia	Caucasian	1037	>70 years	2 years	157
Wennberg et al. [20]	2017	NHATS (the National Health and Aging Trends Study)	USA	Caucasian, African American, Hispanic	7605	>65 years	4 years	4093
Wessels et al. [15]	2011	Diabetes and cognitive decline in elderly African Americans: A 15-year follow-up study	USA	African american	2212	>65 years	15 years	508
Wu et al. [12]	2003	The Sacramento Area Latino Study on Aging (SALSA)	USA	Latino	1789	>60 years	2 years	–
Xu et al. [9]	2010	The Kungsholmen Project	Sweden	Caucasian	1435	>75 years	9 years	235
Yaffe et al. [26]	2012	Health, Aging, and Body composition (Health ABC) Study	USA	African American, Caucasian	3075	>70 years	10 years	6

### Definition of cognitive changes

Studies (see Table 1) meeting the clinical criteria to define cognition and cognitive change were included. There was a large variation between the cognitive tests used. Among the reviewed studies, 35 different tests were used, 22 of which were used in only one study. The Mini Mental State Examination (3MSE/MMSE) was used in nine studies, several fluency tests were used in another nine studies, and the Benton Visual Retention test (BVRT), Wechsler Adult Intelligence Scale (WAIS) and both Immediate and Delayed Recall tests in four studies. Seven different tests or test batteries were used in 2-3 studies. In all studies, the same test battery was completed at baseline and at follow-up. At baseline, higher cognitive scoring showed better cognitive function.

### Quality assessment

All 33 articles were scored using a 12-question checklist (Supplementary file 1) to assess the quality of their contents. One question was scored ad 0-2 points, while the remainder were scored as 0-1 point; the maximum score was 13 points. Two authors (AM-K, SP) provided independent scoring of the eligible articles. In the event of disagreement, a consensus judgement was reached by the other three authors. All articles that scored over 10 points were included for the systematic review.

## Results

The literature search included 3251 eligible abstracts. After evaluation, 33 articles were selected for further evaluation, according to the study criteria. It resulted in 17 peer-reviewed articles, published between 2003 and 2019. The descriptive characteristics of the included studies are listed in Table 1.

In Table 2, the different tests used in the reviewed studies, as well as the cognitive abilities they measured, are listed. The most commonly used test was MMSE or 3MSE, which tests global cognitive functioning.

**Table 2.** Employed cognitive tests that were used more than once.

Test	Tested cognitive ability	Number of time used	References
MMSE/3MSE	Global cognition	9	Yaffe et al. [26], Hassing et al. [21], Xu et al. [9], Formiga et al. [11], Wu et al. [12], Espeland et al. [16], Mayeda et al. [27], Marseglia et al. [10], Altschul et al. [14]
Category fluency tests	Semantic memory	5	Rouch et al. [7], Samaras et al. [8], Bangen et al. [13], Espeland et al. [16], Okereke et al. [18]
Letter fluency tests	Phonemic memory	4	Rouch et al. [7], Samaras et al. [8], Bangen et al. [13], Espeland et al. [16]
BVRT	Visual perception, Visual memory	4	Rouch et al. [7], Samaras et al. [8], Bangen et al. [13], Espeland et al. [16]
WAIS (OR MODIFIED WAIS)	Verbal comprehension, Perceptual reasoning, Working memory, Processing speed	4	Hassing et al. [21], Samaras et al. [8], Bangen et al. [13], Altschul et al. [14]
Digit span test	Working memory	3	Hassing et al. [21], Logroscino et al. [17], Espeland et al. [16]
DSST	Attention, Psychomotor speed, Executive function	2	Yaffe et al. [26], Rouch et al. [7]
Delayed word list	Delayed recall	2	Logroscino et al. [17], Wu et al. [12]
TMT A	Cognitive processing speed	2	Rouch et al. [7], Samaras et al. [8]
TMT B	Executive functioning	2	Rouch et al. [7], Samaras et al. [8]
The boston naming test	Confrontational word retrieval	2	Samaras et al. [8], Bangen et al. [13]
TICS	Global cognition	2	Logroscino et al. [17], Okereke et al. [18]
10-word immediate and delayed recall test	Episodic memory	2	Pappas et al. [19], Wennberg et al. [20]
EBMT	Immediate and delayed recall	2	Logroscino et al. [17], Okereke et al. [18]

### Pre-diabetes stages and cognitive changes

The results regarding pre-diabetes and its association with cognitive differences are shown in Table 3. We included the results of mean cognitive change with *p*-values. Four studies included the available data. Rouch et al. [7] reported pre-diabetes to be linked to a decline in letter fluency, which is associated with executive functions and semantic knowledge. Samaras et al. [8] found that impaired fasting glucose had no detrimental effect on cognition over two years. Xu et al. [9] reported that pre-diabetes accelerated the progression from mild cognitive impairment to dementia. Marseglia et al. [10] found that pre-diabetes was independently associated with accelerated cognitive decline

**Table 3.** Pre-diabetes and cognitive changes.

Authors	Prevalence of IFG n (%)	Cognitive test	Cognitive changes Mean (SE); *p* Value
Marseglia et al. [10]	947/ 2746 (34,5 %)	MMSE	−0.06 (-0.10 to −0.02); <0.01
Rouch et al. [7]	26/163 (15.9%)	FCSR	−0.01 (0.10); 0.37 −0.03 (0.15); 0.89 0.03 (0.40); 0.49 0.01 (0.13); 0.31
		BVRT	−0.01 (0.12); 0.76
		TMT A	0.17 (0.38); 0.24
		TMT B	0.09 (0.29); 0.77
		DSST	−0.02 (0.15); 0.39
		Stroop	0.07 (0.20); 0.06
		Category Fluency Test	0.02 (0.18); 0.77
		Letter Fluency Test	−0.01 (0.19); 0.02
Samaras et al. [8]	346/1037 33.3%	WAIS III	Change reported in Z-scores: Global cognition −0.23 (0.04); 0.84 Processing Speed −0.12 (0.05); 0.10 Memory −0.15 (0.04); 0.79 Language −0.24 (0.05); 0.89 Visuospatial performance −0.07 (0.04); 0.94 Executive function −0.26 (0.06); 0.50
		TMT A	
		TMT B	
		Rey Auditory Verbal Learning Test	
		Logical Memory Story A	
		BVRT	
		Category Fluency Test	
		The Boston Naming Test	
		Letter Fluency Test	
Xu et al. [9]	In cognitively intact cohort 30/877 (3.4%), In MCI cohort 16/268 (6%)	MMSE	Incidental dementia in cognitively intact cohort 7/30 (23.3%); p 0.274 Incidental MCI in cognitively intact cohort 6/30 (21.1%) p 0.220 Incidental dementia in MCI cohort 7/16 (38.9%) p 0.221

### Type 2 diabetes and global cognition

#### Type 2 diabetes and other cognitive abilities

Since many different cognitive tests were used in the reviewed studies, we report the results concerning T2DM associations, the most commonly used cognitive tests and changes of cognition separately, with corresponding *p*-values, in Tables 4–9.

**Table 4.** Systematic review of the association between T2D and global cognition and changes of global cognition.

Authors	T2D at intake n (%)	Cognitive test	Cognitive status Mean (SE)			Cognitive changes Mean (SE)			Association (if available) Odds ratio (95% CI)
			Non-DM	DM	*p* Value	Non-DM	DM	*p* Value	
Espeland et al. [16]	179 (8.3%)	Global cognitive function 3MS	96.8 (3.10)	95.7 (4.27)	<0.001	–	0.21 (0.07)	0.005	
Formiga et al. [11]	42 (25.1%)	MEC (MMSE)	30.5 (3)	30.1 (3)	0.38	2.7 (5.0)	3.8 (5.9)	0.25	OR = 1.37 [1.02 − 1.85]
Hassing et al. [21]	36 (13.1%)	MMSE	27.6 (2.4)	27.7 (2.3)	–	−0.34 (0.06)	−0.49 (0.16)	<0.05	
Logroscino et al. [17]	1394 (7.3%)	TICS	33.8 (2.8)	33.2 (2.9)	–	*–*	*–*		OR ^decline^ = 1.52 [1.15, 1.99]
Mayeda et al. [27]	530 (32.4%)	3MSE	*–*	*–*		*–*	*–*		OR ^decline^ = 1.73 [1.39, 2.16] OR ^T2D at intake^ = 1.88 [1.48, 2.38] OR ^incident cases^ = 0.70 [0.36, 1.35]
Okereke et al. [18]	n (men) = 553 (9.4%) n (women) = 405 (6.4%)	TICS	34.3 (2.7) 34.3 (2.7)	33.8 (3.0) 33.8 (2.8)	- -	- -	- -		
Rouch et al. [7]	12 (7%)	FCSR Total immediate recall Sum immediate free recall Delayed free recall Delayed total recall	46.31 (2.60) 31.93 (5.21) 12.31 (2.23) 15.63 (0.98)	44.81 (3.86) 27.15 (6.55) 11.75 (3.45) 15.29 (1.06)	0.15 0.005 0.45 0.33	0.01 (0.05) −0.03 (0.15) −0.01 (0.19) −0.01 (0.07)	0.04 (0.08) 0.05 (0.17) −0.01 (0.20) −0.01 (0.05)	0.19 0.06 0.99 0.24	
Samaras et al. [8]	106 (12%)	Global cognition (Z-score)	−0.68 (0.05)	−0.97 (0.12)	0.048	−0.92 (0.12)	−0.92 (0.06)	0.05	
Wu et al. [12]	585 (32.7%)	Modified MMSE	84.8	82.5	0.002				OR ^decline^ = 1.68 [1.21, 2.34]
Xu et al. [9]	56 (6%)	MMSE	27.6 (1.3)	27.2 (1.3)	0.678	–	–		
Yaffe et al. [26]	717 (32%)	Modified MMSE	90.9 (0.2)	88.8 (0.3)	0.001	−4.5 (0.3)	−6.0 (0.5)	0.008	

**Table 5.** Systematic review of the association between T2D and Category fluency tests and changes of cognition.

Authors	T2D at intake N (%)	Cognitive test	Cognitive status Mean (SE)			Cognitive changes Mean (SE)		
			Non-DM	DM	*p* Value	Non-DM	DM	*p* Value
Bangen et al. [13]	378 (25.3%)	Category fluency	14.8 ± 4.3	13.8 ± 4.0	<0.001	13.0 ± 4.4	12.2 ± 4.1	0.007
Espeland et al. [16]	n = 179 (8.3%)	Category fluency	29.04 (6.24)	27.77 (6.24)	0.009	–	–	
Okereke et al. [18]	n (men) = 553 (9.4%) n (women) = 405 (6.4%)	Category fluency	2.7 ± 2.0 3.0 ± 2.1	2.4 ± 2.1 2.7 ± 2.0	–	- -	- -	
Rouch et al. [7]	12 (7%)	Category fluency	28.9 (7.75)	31.7 (7.63)	0.21	0.01 (0.23)	−0.11 (0.20)	0.09
Samaras et al. [8]	106 (12%)	Category fluency (Z-score)	−1.23 (0.15)	−0.93 (0.15)	0.21	−0.95 (0.06)	−0.71 (0.06)	0.46

**Table 6.** Systematic review of the association between T2D and BVRT and changes of cognition.

Authors	T2D at intake N (%)	Cognitive test	Cognitive status Mean (SE)			Cognitive changes Mean (SE)		
			Non-DM	DM	*p* Value	Non-DM	DM	*p* Value
Bangen et al. [13]	378 (25.3%)	BVRT Recognition Matching	7.0 (2.2) 8.7 (1.8)	6.6 (2.2) 8.3 (2.0)	0.20 0.18	6.8 (2.3) 8.5 (1.8)	6.3 (2.3) 8.0 (2.0)	0.001 <.001
Espeland et al. [16]	179 (8.3%)	BVRT	7.06 (3.71)	7.78 (4.48)	0.02	–	0.16 (0.07)	0.02
Rouch et al. [7]	12 (7%)	BVRT	12.80 (1.66)	12.06 (2.50)	0.14	0.00 (0.16)	0.07 (0.27)	0.15
Samaras et al. [8]	106 (12%)	BVRT (z-score)	−0.92 (0.15)	−1.23 (0.17)	0.21	−0.71 (0.06)	−0.95 (0.06)	0.18

**Table 7.** Systematic review of the association between T2D and Letter fluency tests and changes of cognition.

Authors	T2D at intake n(%)	Cognitive test	Cognitive status Mean (SE)			Cognitive changes Mean (SE)		
			Non-DM	DM	*p* Value	Non-DM	DM	*p* Value
Bangen et al. [13]	*n* = 378 (25.3%)	Letter Fluency	10.1 ± 4.4	8.7 ± 4.0	<0.001	9.8 ± 6.1	8.7 ± 6.2	0.007
Espeland et al. [16]	*n* = 179 (8.3%)	Letter fluency	39.83 (12.54)	37.39 (12.12)	0.01	–	–	–
Rouch et al. [7]	*n* = 36 (22 %)	Letter Fluency	17.65 (6.73)	17.66 (8.11)	0.99	0.23 (0.45)	0.24 (0.42)	0.93
Samaras et al. [8]	*n* = 511 (49.3 %)	Language (z-score)	−1.23 (0.15)	−0.93 (0.15)		−0.95 (0.06)	−0.71 (0.06)	0.46

**Table 8.** Systematic review of the association between T2D and WAIS and changes of cognition.

Authors	T2D at intake N (%)	Cognitive test	Cognitive status Mean (SE)			Cognitive changes Mean (SE)		
			Non-DM	DM	*p* Value	Non-DM	DM	*p* Value
Bangen et al. [13]	378 (25.3%)	Similarities subtest of the WAIS	12.0 ± 7.3	9.5 ± 7.1	<0.001	11.8 ± 7.6	9.6 ± 7.2	<0.01
Hassing et al. [21]	36 (13.1%)	Modified WAIS (symbol digit substitution test)	26.0 ± 11.5	24.1 ± 11.6		−0.55 (0.11)	−0.68 (0.31)	<0.05
Samaras et al. [8]	106 (12%)	WAIS III	−0.39 (0.05)	−0.62 (0.11)	0.09	−0.39 (0.05	−0.91 (0.16)	0.27

**Table 9.** Systematic review of the association between T2D and DST and changes of cognition.

Authors	T2D at intake N (%)	Cognitive test	Cognitive status Mean (SE)			Cognitive changes Mean (SE)		
			Non-DM	DM	*p* Value	Non-DM	DM	*p* Value
Espeland et al. [16]	179 (8.3%)	Digit Span Forward Digit Span Backward	7.45 (2.09) 6.67 (2.02)	7.33 (1.87) 6.72 (2.09)	0.45 0.73	Change in cognition adjusted for all risk factors, attention/working memory 0.04 (0.07) p 0.56		
Hassing et al. [21]	36 (13.1%)	Digit Span Forward Digit Span Backward	5.5 ± 1.2 3.5 ± 1.6	5.5 +/. 1.4 3.5 ± 1.2		−0.10 (0.2) −0.11 (0.02)	−0.02 (0.04) −0.08 (0.05)	<0.05 <0.05
Logroscino et al. [17]	1394 (7.3%)	Digit span Backwards	6.7 (2.4)	6.4 (2.4)		The change was reported in global cognition score		

Eleven of the 17 studies measured global cognition scores, but the tests themselves varied (Table 4). Five out of the nine studies that used MMSE for the assessment of cognition found statistically significant associations. There was a clear tendency towards increased cognitive decline in older people with T2DM, albeit without statistical significance [11,12] . A wider test battery did not result in statistically significant association between T2DM and cognitive decline [7,13] , although T2DM was associated with worse baseline cognitive performance [13]. Altschul et al. [14] used several tests and reported the change of cognition as overall cognitive function, and they additionally found that A1C from age 70 to 79 did not have any consistent association with change in cognitive function. Wessels et al. [15] also used a wide test battery (but not MMSE) and found that diabetes was associated with cognitive decline.

Category fluency tests, which measure semantic memory, were used in five studies (Table 5). Espeland et al. [16], Logroscino et al. [17] and Okereke et al. [18] did not report the change of cognition in category fluency test separately, but rather as part of a change in total verbal fluency or global cognition. Bangen et al. [13] did not report a change in cognitive test results; instead, they gave the test results at the follow-up point. None of the studies found a statistically significant association between T2DM and cognitive change during aging when a category fluency test was used.

A letter fluency tests were used as a separate test in four studies (Table 7). These tests are ordinarily used to assess phonemic memory. Changes were reported in two of the four studies. A statistically significant change was not reported when using a letter fluency test.

Pappas et al. [19] used immediate and delayed list recall (episodic memory), and they found that T2DM was associated with a decline in episodic memory. Also, Wennberg et al. [20] found the same association using immediate and delayed recall word-list learning tests.

Four studies used BVRT, which is employed to assess visual perception and visual memory (Table 6). Bangen et al. [13] did not report a change in test results. Only Espeland et al. [16] found a statistically significant association when BVRT was used.

WAIS consists of four indices: verbal comprehension, perceptual reasoning, working memory and processing speed. Four of 17 studies used WAIS or its modified version for cognitive assessment (Table 8). Hassing et al. [21] reported a change of cognition, with a statistically significant association between diabetes and cognitive change. Two [8,13] of the studies did not find a statistically significant association. Altschul et al. [14] did not report the result for this test separately.

The Digit Span Test was used in three studies (Table 9) and Hassing et al. [21] reported a statistically significant change in cognition. Espeland et al. [16] found no statistically significant association between T2DM and cognitive change with this test. Logroscino et al. [17] did not report the results for this test separately.

## Discussion

Basic and clinical research support a link between T2DM and Alzheimer's disease (AD), although the relationship with Alzheimer's Disease progression remains unclear [22]. It has also been suspected that an association between diabetes and cognitive decline during aging might exist. Pre-diabetes has also been hypothesized as a risk for cognitive decline. In the present review, we aimed to analyze whether a link could be systematically observed between cognitive changes and glucose disorders among persons with normal cognition, but without diagnosed cognitive disease.

Most of the reviewed studies had considered diagnosed T2DM and its association to cognition. Few, however, also studied the pre-diabetes stages. Almost all the studies found a tendency for T2DM or pre-diabetes to be a risk for weaker cognitive functioning, although the statistical significance was not clear in every study.

The prevalence of T2DM varied widely between 17 reviewed studies. The size of the diabetes group did not seem to affect the result, as some of the studies with relatively low prevalence found a statistically significant association between T2DM and cognitive decline. On the other hand, for example, Wu et al. [12] had a high prevalence of T2DM (32,7%) in their sample, yet a statistically significant association was not found.

The follow up times ranged between 2 to 15 years among the reviewed studies, and the length of the follow-up time did not seem to affect the result. Ten studies had a follow-up time of six or more years. Five out of 10 studies with a longer follow-up time and four out of seven studies with a shorter follow-up time did not find a statistically significant association with T2DM and cognitive decline.

It seemed that the wider the test battery was, the more obvious the effect of glucose disorders to cognitive decline during aging. The most used test among now reviewed articles was MMSE. In a systematic review and meta-analysis in 2015, Tsoi et al. [23] examined 102 studies that included 36,080 participants in order to evaluate the diagnostic performance of MMSE. They demonstrated that MMSE is the most frequently studied test for dementia screening, and the combined sensitivity and specificity of MMSE for the detection of dementia was 0.81 (95% CI, 0.78-0.84) and 0.89 (95% CI, 0.87-0.91), respectively. MMSE (or 3MSE) is used in screening of Alzheimer’s disease, but it alone is insufficient to exclude incipient dementia. A review in 2017 concluded that recall tests were the most effective test for mild cognitive impairment (MCI) detection, and that these tests can potentially be used as a triage screening test for MCI in primary care settings; but when a patient shows cognitive impairments beyond memory deterioration, a more comprehensive test should be used [24]. Li et al. found that MMSE and word fluency test showed significant changes only in Alzheimer’s Disease participants, not among those who have mild cognitive impairment [25]. Thus, it is obvious that a wider test battery and a more comprehensive test would identify cognitive domains other than memory, and that the possibility for cognitive change (decline) during follow-up is more evident.

Among the 17 now reviewed studies, nine [9,10,11,12,14,16,21,26,27] used MMSE or 3MSE for evaluation of cognitive functioning and as a follow-up test (Table 2). Hassing et al. [21] used 11, while Espeland et al. [16] used seven, different cognitive tests during the follow-up, and both studies found a statistically significant association between T2DM and cognitive decline. Seven studies [9,10,11,12,14,26,27] used only MMSE/3MSE or MMSE and 1-2 other test, four [11,12,14,26] found no significant association between T2DM and cognitive decline. Mayeda et al. [27] found that baseline T2DM was associated with cognitive decline, whereas new-onset T2DM was not. As stated previously, Xu et al. [9] found that both T2DM and pre-diabetes accelerate the progression from mild cognitive impairment to dementia, but they found no significant association of either T2DM or pre-diabetes with incident MCI with cognitively intact persons. Marseglia et al. [10] found that both pre-diabetes and T2DM were associated with cognitive decline.

The strength of this review is that many different cognitive tests were used among the studies; and even though it made the results difficult to compare, we were nonetheless able to evaluate many tests that measure different cognitive domains. On the other hand, most of the studies used MMSE, which in turn gave us the opportunity to compare the results of several studies that used the same test. When global cognition was assessed, a significant association was found more often, whereas tests measuring some focal cognitive ability did not necessarily find a statistical significance. There are only a few studies about this subject among elderly, and therefore we believe that the importance of this review is that it focused on studies among individuals over 65 years of age, thereby offering new information about the importance of preventing glucose metabolism disorders in older age especially.

There are some limitations in this review. We did not find as strong an association as we hypothesized, and this may be due to several factors. First, several different cognitive tests or test batteries were used, which in turn seemed to affect the results such that wider testing often gave a significant association. Quite surprisingly, the length of the follow-up time did not seem to be such an important factor, although Wu et al. [12] for example, suspected their follow-up time to be too short to find statistically significant association between T2DM and cognitive decline during aging. Generally, it is known that there are differences between men and women in morbidity and health behavior, but sex-specific impacts were missing among the studies. One study was from Australia and all others from Europe or USA. So, there is a lack of studies from other parts of the world and it can be discussed whether the associations may vary among populations, ethnicities and cultures. In addition, even when the same standard test was used, the results or scoring could be different if, for example, a modified version of the test was used. The studies also reported the results in different ways – for example, Samaras et al. [8] used z- scoring, while most commonly the test results were reported as an exact numerical value. For these reasons, the comparison of the results was not always simple.

## Conclusion

This review focused in the studies regarding the association between cognitive changes and prediabetes/T2DM and we found that the results are heterogeneous. Eleven of the 17 reviewed studies found a statistically significant decline in cognition among individuals who had T2DM compared to their non-diabetic references. Only four studies had considered prediabetes and cognitive changes and three of them found a statistically significant association. This gives in percent a high result, but the sampling size is relatively small. According to former cross-sectional studies our hypothesis was that the connection would be evident, and we expected that a clear majority of the studies would find statistically significant association also in longitudinal setting. Relative to that hypothesis, our result that 65% of the studies concerning T2DM found a statistically significant decline seems lower than expected.

In clinical aspect, further research is needed to evaluate whether it would be possible to prevent cognitive changes and promote healthy aging by protecting individuals from glucose metabolism disorders, as well as how to better prevent pre-diabetes and T2DM stages among elderly without a diagnosed memory disorder. More investigation on the effect of glucose metabolism disorders in normal aging is needed to determine whether T2DM and pre-diabetes accelerate cognitive aging independently.
